# 5-(3-Fluoro­phen­yl)-1,3,4-thia­diazol-2-amine

**DOI:** 10.1107/S1600536809019333

**Published:** 2009-05-29

**Authors:** Yao Wang, Rong Wan, Feng Han, Peng Wang

**Affiliations:** a Department of Applied Chemistry, College of Science, Nanjing University of Technology, No. 5 Xinmofan Road, Nanjing 210009, People’s Republic of China

## Abstract

The title compound, C_8_H_6_FN_3_S, was synthesized by the reaction of 3-fluoro­benzoic acid and thio­semicarbazide. The dihedral angle between the planes of the thia­diazole and benzene rings is 37.3 (2)°. In the structure, two crystallographically independent mol­ecules form a centrosymmetric dimer, in which two inter­molecular N—H⋯N hydrogen bonds generate an *R*_2_^2^(8) motif.

## Related literature

For the biological activity of 1,3,4-thiadiazole derivatives, see: Nakagawa *et al.* (1996 ▶); Wang *et al.* (1999 ▶). For a similar structure, see: Wan *et al.* (2006 ▶). For bond-length data, see: Allen *et al.* (1987 ▶).
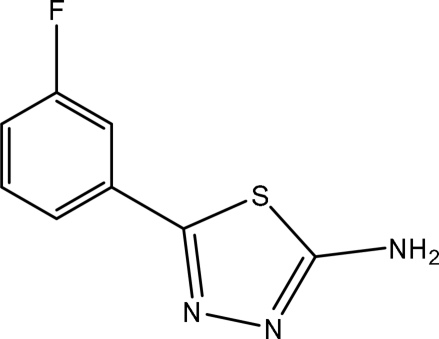


## Experimental

### 

#### Crystal data

C_8_H_6_FN_3_S
*M* *_r_* = 195.23
Monoclinic, 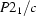

*a* = 11.345 (2) Å
*b* = 7.3130 (15) Å
*c* = 11.269 (2) Å
β = 111.64 (3)°
*V* = 869.0 (3) Å^3^
*Z* = 4
Mo *K*α radiation
μ = 0.34 mm^−1^
*T* = 293 K
0.20 × 0.10 × 0.10 mm

#### Data collection

Enraf–Nonius CAD-4 diffractometer
Absorption correction: ψ scan (North *et al.*, 1968 ▶) *T*_min_ = 0.935, *T*_max_ = 0.967
1667 measured reflections
1584 independent reflections
1177 reflections with *I* > 2σ(*I*)
*R*_int_ = 0.021
3 standard reflections every 200 reflections intensity decay: 1%

#### Refinement

*R*[*F* ^2^ > 2σ(*F* ^2^)] = 0.047
*wR*(*F* ^2^) = 0.124
*S* = 1.01
1584 reflections
118 parameters
13 restraints
H-atom parameters constrained
Δρ_max_ = 0.37 e Å^−3^
Δρ_min_ = −0.25 e Å^−3^

### 

Data collection: *CAD-4 Software* (Enraf–Nonius, 1989 ▶); cell refinement: *CAD-4 Software*; data reduction: *XCAD4* (Harms & Wocadlo, 1995 ▶); program(s) used to solve structure: *SHELXS97* (Sheldrick, 2008 ▶); program(s) used to refine structure: *SHELXL97* (Sheldrick, 2008 ▶); molecular graphics: *SHELXTL* (Sheldrick, 2008 ▶); software used to prepare material for publication: *SHELXL97*.

## Supplementary Material

Crystal structure: contains datablocks global, I. DOI: 10.1107/S1600536809019333/at2787sup1.cif

Structure factors: contains datablocks I. DOI: 10.1107/S1600536809019333/at2787Isup2.hkl

Additional supplementary materials:  crystallographic information; 3D view; checkCIF report

## Figures and Tables

**Table 1:** Hydrogen-bond geometry (Å, °)

*D*—H⋯*A*	*D*—H	H⋯*A*	*D*⋯*A*	*D*—H⋯*A*
N3—H3*A*⋯N2^i^	0.86	2.14	2.981 (5)	165

Symmetry code: (i) 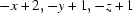.

**Fig. 1.**
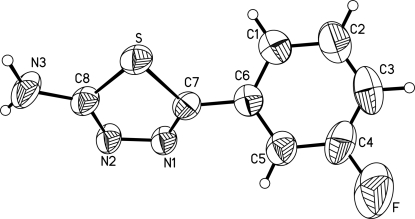
A view of the molecular structure of (I). Displacement ellipsoids are drawn at the 50% probability level.

**Fig. 2.**
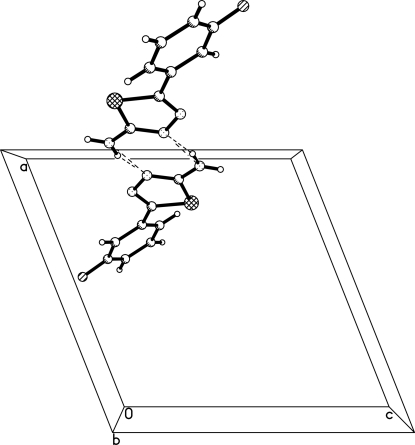
A view of a dimer structure, in which two intermolecular N—H···N hydrogen bonds generate a motif R22(8).

## supplementary crystallographic information

### Comment

1,3,4-Thiadiazole derivatives represent an interesting class of compounds possessing broad spectrum biological activities (Nakagawa *et al.*, 1996). These compounds are known to exhibit diverse biological effects, such as insecticidal, fungicidal activities (Wang *et al.*, 1999). We are focusing our synthetic and structural studies on thiadiazole derivatives and have published the structure of 5-(4-fluoro-phenyl)-[1,3,4]thiadiazol-2-ylamine (Wan *et al.*, 2006). Here we report the crystal structure, (I).

In the title molecule (I), (Fig. 1), the dihedral angle between the thiadiazole and benzene ring is 37.3 (2)°, which is bigger than the angle in the structure of 5-(4-fluoro-phenyl)-[1,3,4]thiadiazol-2-ylamine (Wan *et al.*, 2006), which is 30.1 (2)°. In the structure, two crystallographically independent molecules form a dimer structure, in which two intermolecular N—H···N hydrogen bonds generate a motif *R*_2_^2^(8) (Fig. 2).

### Experimental

3-Fluoro-benzoic acid (2 mmol) and thiosemicarbazide (5 mmol) were mixed in a 25 ml flask, and kept in the oil bath at 363 K for 6 h. After cooling, the crude product (I) precipitated and was filted. Pure compound (I) was obtained by crystallization from ethanol (20 ml). Crystals of (I) suitable for X-ray diffraction were obtained by slow evaporation of an acetone solution.

### Refinement

All H atoms were placed geometrically with C—H = 0.93 Å and N—H = 0.86 Å, and included in the refinement in riding motion approximation with *U*_iso_(H) = 1.2*U*_eq_ of the carrier atom.

### Crystal data

**Table:**

C_8_H_6_FN_3_S	*F*(000) = 400
*M**_r_* = 195.23	*D*_x_ = 1.492 Mg m^−^^3^
Monoclinic, *P*2_1_/*c*	Melting point: 515 K
Hall symbol: -P 2ybc	Mo *K*α radiation, λ = 0.71073 Å
*a* = 11.345 (2) Å	Cell parameters from 25 reflections
*b* = 7.3130 (15) Å	θ = 10–13°
*c* = 11.269 (2) Å	µ = 0.34 mm^−^^1^
β = 111.64 (3)°	*T* = 293 K
*V* = 869.0 (3) Å^3^	Block, colourless
*Z* = 4	0.20 × 0.10 × 0.10 mm

### Data collection

**Table:**

Enraf–Nonius CAD-4 diffractometer	1177 reflections with *I* > 2σ(*I*)
Radiation source: fine-focus sealed tube	*R*_int_ = 0.021
graphite	θ_max_ = 25.3°, θ_min_ = 1.9°
ω/2θ scans	*h* = −13→0
Absorption correction: ψ scan (North *et al.*, 1968)	*k* = 0→8
*T*_min_ = 0.935, *T*_max_ = 0.967	*l* = −12→13
1667 measured reflections	3 standard reflections every 200 reflections
1584 independent reflections	intensity decay: 1%

### Refinement

**Table:**

Refinement on *F*^2^	Primary atom site location: structure-invariant direct methods
Least-squares matrix: full	Secondary atom site location: difference Fourier map
*R*[*F*^2^ > 2σ(*F*^2^)] = 0.047	Hydrogen site location: inferred from neighbouring sites
*wR*(*F*^2^) = 0.124	H-atom parameters constrained
*S* = 1.01	*w* = 1/[σ^2^(*F*_o_^2^) + (0.06*P*)^2^] where *P* = (*F*_o_^2^ + 2*F*_c_^2^)/3
1584 reflections	(Δ/σ)_max_ < 0.001
118 parameters	Δρ_max_ = 0.37 e Å^−^^3^
13 restraints	Δρ_min_ = −0.25 e Å^−^^3^

### Special details

**Table:**

Geometry. All e.s.d.'s (except the e.s.d. in the dihedral angle between two l.s. planes) are estimated using the full covariance matrix. The cell e.s.d.'s are taken into account individually in the estimation of e.s.d.'s in distances, angles and torsion angles; correlations between e.s.d.'s in cell parameters are only used when they are defined by crystal symmetry. An approximate (isotropic) treatment of cell e.s.d.'s is used for estimating e.s.d.'s involving l.s. planes.
Refinement. Refinement of *F*^2^ against ALL reflections. The weighted *R*-factor *wR* and goodness of fit *S* are based on *F*^2^, conventional *R*-factors *R* are based on *F*, with *F* set to zero for negative *F*^2^. The threshold expression of *F*^2^ > σ(*F*^2^) is used only for calculating *R*-factors(gt) *etc*. and is not relevant to the choice of reflections for refinement. *R*-factors based on *F*^2^ are statistically about twice as large as those based on *F*, and *R*- factors based on ALL data will be even larger.

### Fractional atomic coordinates and isotropic or equivalent isotropic displacement parameters (Å^2^)

**Table:**

	*x*	*y*	*z*	*U*_iso_*/*U*_eq_	
S	0.82144 (7)	0.08639 (11)	0.55908 (6)	0.0548 (3)	
F	0.5162 (3)	−0.2428 (3)	0.0198 (2)	0.1125 (9)	
N1	0.8658 (2)	0.1443 (3)	0.3566 (2)	0.0523 (6)	
C1	0.7322 (3)	−0.2918 (5)	0.3984 (3)	0.0713 (9)	
H1B	0.7804	−0.3036	0.4850	0.086*	
N2	0.9262 (2)	0.2841 (3)	0.4381 (2)	0.0551 (6)	
C2	0.6592 (4)	−0.4354 (5)	0.3322 (4)	0.0817 (11)	
H2B	0.6585	−0.5443	0.3746	0.098*	
N3	0.9606 (3)	0.3931 (4)	0.6429 (2)	0.0733 (9)	
H3A	1.0043	0.4846	0.6340	0.088*	
H3B	0.9481	0.3787	0.7131	0.088*	
C3	0.5871 (4)	−0.4202 (5)	0.2040 (4)	0.0822 (12)	
H3C	0.5378	−0.5175	0.1592	0.099*	
C4	0.5898 (3)	−0.2597 (6)	0.1447 (3)	0.0741 (10)	
C5	0.6637 (3)	−0.1132 (4)	0.2071 (3)	0.0639 (9)	
H5A	0.6658	−0.0066	0.1628	0.077*	
C6	0.7338 (3)	−0.1272 (4)	0.3350 (3)	0.0516 (7)	
C7	0.8082 (3)	0.0307 (4)	0.4039 (2)	0.0475 (7)	
C8	0.9121 (3)	0.2733 (4)	0.5476 (2)	0.0499 (7)	

### Atomic displacement parameters (Å^2^)

**Table:**

	*U*^11^	*U*^22^	*U*^33^	*U*^12^	*U*^13^	*U*^23^
S	0.0686 (5)	0.0626 (5)	0.0402 (4)	−0.0130 (4)	0.0282 (3)	0.0002 (3)
F	0.1200 (19)	0.122 (2)	0.0888 (16)	−0.0253 (16)	0.0307 (14)	−0.0193 (15)
N1	0.0646 (15)	0.0583 (14)	0.0421 (12)	−0.0113 (12)	0.0293 (11)	−0.0079 (11)
C1	0.070 (2)	0.068 (2)	0.073 (2)	−0.0121 (18)	0.0235 (17)	0.0023 (18)
N2	0.0753 (16)	0.0580 (15)	0.0433 (12)	−0.0172 (13)	0.0353 (12)	−0.0097 (11)
C2	0.074 (2)	0.060 (2)	0.112 (3)	−0.0081 (19)	0.035 (2)	0.001 (2)
N3	0.110 (2)	0.0800 (19)	0.0452 (14)	−0.0379 (17)	0.0462 (15)	−0.0191 (13)
C3	0.074 (2)	0.079 (3)	0.104 (3)	−0.020 (2)	0.045 (2)	−0.040 (2)
C4	0.075 (2)	0.091 (3)	0.062 (2)	−0.024 (2)	0.0319 (18)	−0.0344 (19)
C5	0.075 (2)	0.068 (2)	0.0514 (17)	−0.0144 (17)	0.0271 (16)	−0.0140 (16)
C6	0.0557 (17)	0.0533 (17)	0.0519 (16)	0.0000 (13)	0.0270 (14)	−0.0051 (13)
C7	0.0541 (16)	0.0513 (16)	0.0421 (14)	0.0000 (13)	0.0236 (13)	−0.0013 (12)
C8	0.0634 (18)	0.0556 (17)	0.0374 (13)	−0.0068 (14)	0.0265 (13)	−0.0025 (13)

### Geometric parameters (Å, °)

**Table:**

S—C8	1.744 (3)	C2—H2B	0.9300
S—C7	1.747 (3)	N3—C8	1.338 (3)
F—C4	1.351 (4)	N3—H3A	0.8600
N1—C7	1.288 (3)	N3—H3B	0.8600
N1—N2	1.377 (3)	C3—C4	1.357 (5)
C1—C2	1.375 (5)	C3—H3C	0.9300
C1—C6	1.403 (4)	C4—C5	1.382 (4)
C1—H1B	0.9300	C5—C6	1.369 (4)
N2—C8	1.303 (3)	C5—H5A	0.9300
C2—C3	1.378 (5)	C6—C7	1.472 (4)
			
C8—S—C7	86.76 (13)	F—C4—C3	118.3 (3)
C7—N1—N2	114.0 (2)	F—C4—C5	119.0 (4)
C2—C1—C6	119.8 (3)	C3—C4—C5	122.7 (3)
C2—C1—H1B	120.1	C6—C5—C4	119.0 (3)
C6—C1—H1B	120.1	C6—C5—H5A	120.5
C8—N2—N1	112.5 (2)	C4—C5—H5A	120.5
C1—C2—C3	120.9 (4)	C5—C6—C1	119.3 (3)
C1—C2—H2B	119.6	C5—C6—C7	119.6 (3)
C3—C2—H2B	119.6	C1—C6—C7	121.1 (3)
C8—N3—H3A	120.0	N1—C7—C6	124.6 (2)
C8—N3—H3B	120.0	N1—C7—S	113.2 (2)
H3A—N3—H3B	120.0	C6—C7—S	122.1 (2)
C4—C3—C2	118.3 (3)	N2—C8—N3	124.3 (3)
C4—C3—H3C	120.9	N2—C8—S	113.5 (2)
C2—C3—H3C	120.9	N3—C8—S	122.19 (19)
			
C7—N1—N2—C8	−0.2 (4)	N2—N1—C7—S	0.5 (3)
C6—C1—C2—C3	−0.1 (5)	C5—C6—C7—N1	−36.0 (4)
C1—C2—C3—C4	−0.1 (6)	C1—C6—C7—N1	144.6 (3)
C2—C3—C4—F	−178.1 (3)	C5—C6—C7—S	141.4 (3)
C2—C3—C4—C5	1.3 (6)	C1—C6—C7—S	−37.9 (4)
F—C4—C5—C6	177.0 (3)	C8—S—C7—N1	−0.4 (2)
C3—C4—C5—C6	−2.4 (5)	C8—S—C7—C6	−178.2 (2)
C4—C5—C6—C1	2.2 (5)	N1—N2—C8—N3	−179.5 (3)
C4—C5—C6—C7	−177.2 (3)	N1—N2—C8—S	−0.1 (3)
C2—C1—C6—C5	−1.0 (5)	C7—S—C8—N2	0.3 (2)
C2—C1—C6—C7	178.4 (3)	C7—S—C8—N3	179.7 (3)
N2—N1—C7—C6	178.1 (2)		

### Hydrogen-bond geometry (Å, °)

**Table:**

*D*—H···*A*	*D*—H	H···*A*	*D*···*A*	*D*—H···*A*
N3—H3A···N2^i^	0.86	2.14	2.981 (5)	165

Symmetry codes: (i) −*x*+2, −*y*+1, −*z*+1.
